# Disposable Copper Electrodes Based on Printed Circuit Board Technology for the *Operando* Generation of Raman-Enhancing Substrates

**DOI:** 10.1021/acs.analchem.5c02381

**Published:** 2025-09-16

**Authors:** Martin Perez-Estebanez, Luis Romay, Maria Huidobro, Pello Nuñez-Marinero, Aranzazu Heras, F. Javier del Campo, Alvaro Colina

**Affiliations:** † Department of Chemistry, Universidad de Burgos, Pza. Misael Bañuelos s/n, E-09001 Burgos, Spain; ‡ BCMaterials, Basque Center for Materials, Applications and Nanostructures, UPV/EHU Parque Científico, E-48940 Leioa, Bizkaia ,Spain

## Abstract

Most surface-enhanced Raman scattering (SERS) substrates reported in the literature consist of plasmonic nanostructures, mainly based on gold or silver. Although Cu plasmonic properties have been known for decades, Cu-SERS substrates remain limited by their limited chemical stability. Nevertheless, many researchers claim that Cu-based SERS substrates should be revisited, as new synthetic strategies have led to the improved stability of Cu nanostructures. Among these, the electrosynthesis of Cu nanomaterials has been proposed as an interesting approach because it allows the in situ generation of SERS substrates. Additionally, the combination of electrochemistry with *operando* Raman spectroscopy, also known as Raman spectroelectrochemistry, provides real-time information about the electrogenerated SERS substrates. In this work, we report the use of Cu-printed circuit boards to create an electrochemical platform, which can be used for the *operando* generation of Cu-based SERS substrates in chloride media. These SERS substrates exhibit great potential for the detection of tertiary amines such as cocaine, fentanyl, paraquat, or adenine. A complementary study using bidimensional UV/vis absorption spectroelectrochemistry was carried out to fully understand the processes occurring during the *operando* generation of the Cu-SERS substrates.

## Graphical abstract

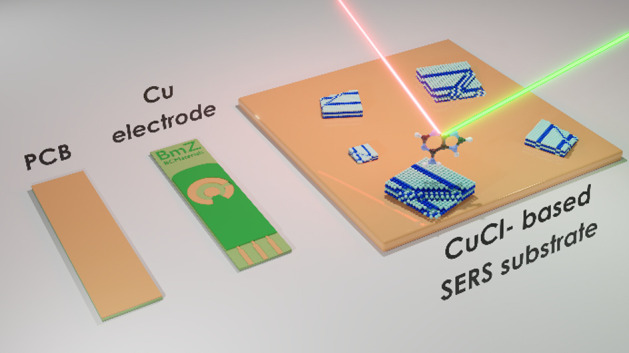

## 1 Introduction

Copper is of great interest in many fields, from catalysis,^1,2^ to the development of Surface-Enhanced Raman Scattering (SERS) substrates.^3^ Cu, Ag and Au are typically used as SERS substrates^4,5^ thanks to their plasmonic properties. However, among these plasmonic metals, Cu is the least used due to its low chemical stability.^6^ Nevertheless, a significant number of studies have demonstrated the remarkable potential of Cu in the generation of SERS substrates and electrochemically assisted SERS (EC-SERS) substrates,^3,6–8^ suggesting that this metal should be reconsidered as a powerful candidate for the generation of plasmonic substrates.

Cu is also an interesting material from a manufacturing perspective, since it is a cheap and abundant element in the Earth’s crust, especially when compared to Ag and Au.^6^ Additionally, Cu is extensively used in industry worldwide. One of its most important applications is in the manufacturing of Printed Circuit Board (PCB) plates, commonly used in the production of electronic components.^9^ Cu-PCB plates consist of a thick plate of electrodeposited Cu on an inert substrate (FR4), which allows for the inexpensive and simple manufacture of Cu printed circuits boards. In this work, we explore the use of PCB technology for the fabrication of Cu-based Printed Circuit Electrodes (Cu-PCE), useful for EC-SERS measurements.

Typical strategies for generating Cu electrodes (for electrochemical or SERS measurements) rely on chemical or physical vapor deposition (CVD, PVD) methodologies.^10,11^ Although these techniques are powerful manufacturing technologies, they are associated with high energy consumption, and are mainly optimized for the deposition of thin films, usually with nanometric thickness.^12^ The use of PCB for electrode manufacturing is an attractive alternative to CVD or PVD, since it allows an easy, inexpensive and scalable method to manufacture electrodes. PCB plates can be manufactured at a cost as low as $0.20 per cm^2^, thanks to the maturity of PCB industry.^9^ PCB technology also allows easy customization of circuit geometry for the design of sophisticated prototypes, useful for development of consumer electronics, including point-of-care devices.^13,14^

Cu-PCB electrodes can be further modified by wet chemistry, for example, through electroless deposition of Au or Ag coatings.^13,15^ This strategy has been widely used to create various electrochemical setups; however, only a few studies have been published using unmodified PCBs as working electrode (WE).^16,17^ To the best of our knowledge, this is the first report on the use of Cu-PCE as EC-SERS substrates.

The main objective of this study is to develop a Cu-PCE that combines the advantages of classical screen-printed electrodes (SPEs), such as reproducibility, disposability, and ease of manufacture, while overcoming their limitations, such as the introduction of uncontrolled impurities from screen-printing inks into the WE. It will be demonstrated that commercially available Cu-PCB plates can be used to generate a variety of very interesting Raman-enhancing substrates that can be easily used to perform *operando* Raman spectroelectrochemistry (Raman-SEC) experiments.

## 2 Methods and Materials

### 2.1 Chemical Reagents

Hydrochloric acid (HCl, 37%, VWR), adenine (>99%, Sigma-Aldrich), cocaine hydrochloride (>97.5%, Sigma), fentanyl (Ann Arbor, MI), and paraquat (96%, Aldrich) were used as received, without further purification. All solutions were prepared using ultrapure water from a Millipore SQ 2 Series (18.2 MΩ cm resistivity at 25 °C).

### 2.2 Instrumentation

In situ time-resolved Raman SEC (TR-Raman-SEC) experiments were performed using a customized SPELEC-RAMAN instrument (Metrohm-DropSens), which included a 785 nm laser source, a proper spectrophotometer, and a potentiostat. Laser power was fixed for all experiments to 102 mW, (325 W cm^–2^). Bidimensional UV/vis absorption SEC (BSEC) experiments were performed using a reflection probe (2.5 mm in diameter, Avantes) protected with Mylar and focused on the WE for the normal configuration. Measurements in parallel configuration were performed using two bare optical fibers (100 μm in diameter, Ocean Optics) glued on a PMMA piece, as reported in previous works.^18^ BSEC experiments were carried out using two customized SPELEC instruments (Metrohm-DropSens). Each customized SPELEC equipment included a potentiostat and a suitable spectrophotometer, using the same halogen-deuterium lamp in both configurations. All equipment components were properly synchronized, ensuring the accurate correlation of the electrochemical and spectroscopic data, as well as reliable *operando* and time-resolved data acquisition. All measurements were performed using the DropView software (Metrohm-DropSens).

A Zeiss GeminiSEM560 field-emission scanning electron microscope (FE-SEM) was used to obtain the SEM images of the WE surface. An electron beam of 2 kV was used, with an in-lens secondary-electron detector. This FE-SEM equipment integrated an EDX analyzer, which was used to carry out EDX mapping. An electron beam of 6 kV was used for EDX mapping.

## 3 Results and Discussion

### 3.1 Design and Fabrication of Cu-PCE

In this work, the use of PCB technology for the fabrication of whole-Cu Printed Circuit Electrodes (Cu-PCE) is proposed. The manufacturing process is shown in Figure 1A and described in the Supporting Information (SI). Cu-PCE fabrication starts with the initial cleaning of a bare, commercial Cu-PCB plate by sonication in consecutive baths of acetone, isopropanol and deionized water (a). Electrode geometry was defined by screen printing of a thermocurable resist (b), and then immersed in a FeCl_3_ bath to remove all exposed Cu on the plate by chemical etching, leaving only the metal coated with the photoresistor insulator (c). Next, the resist was stripped by immersion in a 5% NaOH solution (d). Finally, a dielectric polymer was screen-printed on the Cu surface to define the Cu active area conforming to the electrochemical system, where the three electrodes, WE, counter electrode (CE) and pseudo reference electrode (RE) are made of Cu (e).

**Figure 1:**
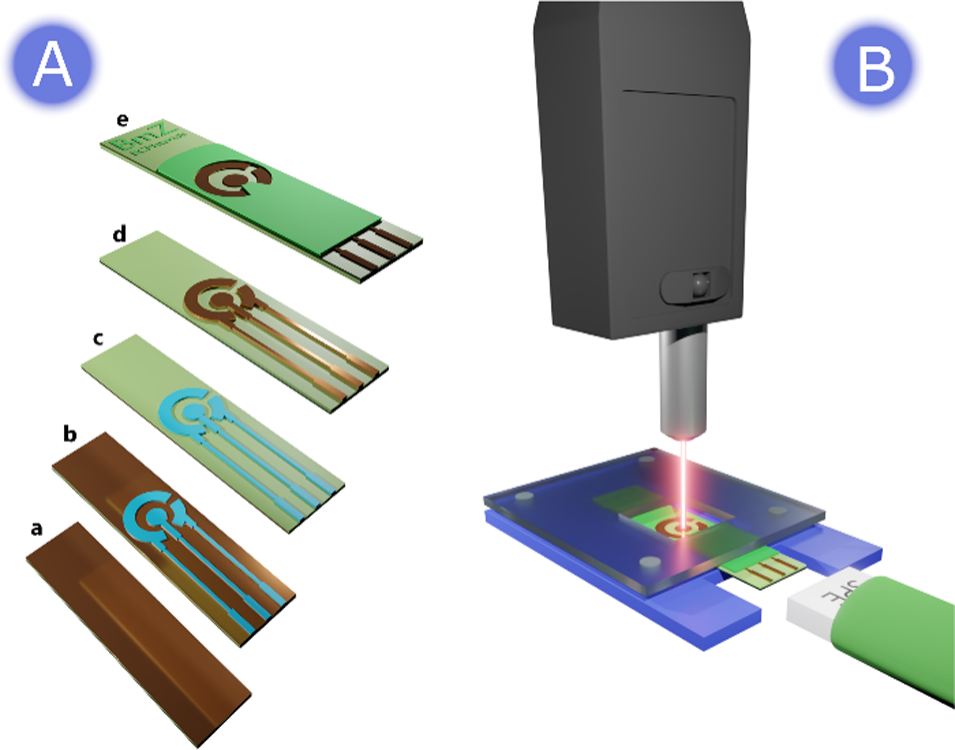
(A) Description of the Cu-PCE manufacturing process: bare PCB plate (a), after applying a photoresistor (b), after chemical etching of Cu with a FeCl_3_ bath (c), after removing photoresistor (d), and after screen-printing the dielectric layer (e). (B) Schematic of the experimental setup used for Raman-SEC experiments.

This strategy allows the fabrication of an electrochemical platform made of three whole-Cu electrodes acting as WE, RE and CE, which can be used for both electrochemical and spectroelectrochemical measurements. These Cu-PCEs were proved stable for electrochemical applications. As a proof of concept, 5 cyclic voltammograms were performed in NaOH, demonstrating the good electrochemical performance of these substrates for long-term experiments (Figure S1). In this experiment, the Cu-RE remained stable for several cycles. If required, several strategies have been reported to further stabilize the Cu-RE, such as covering it with insoluble copper chloride.^11^

### 3.2 Cu-PCEs for EC-SERS Measurements

Next, the performance of the prepared Cu-PCEs as platforms for the generation of EC-SERS substrates was evaluated by cyclic voltammetry Raman-SEC experiments, where metallic Cu nanoparticles (CuNPs) were synthesized by oxidation–reduction cycles (ORC) of the electrode in the presence of adenine (Figure 2). Cyclic voltammetry was carried out at a Cu-PCE at 0.02 V/s from −0.60 and +0.40 V (vs Cu), starting at −0.20 V toward positive potentials, in 8 μM adenine and 0.1 M HCl. Experiments were conducted at pH = 1 to avoid the formation of Cu oxides. The corresponding cyclic voltammogram (CV) is shown in Figure 2A (blue line). Cu electro-oxidation in chloride media has been previously described using electrochemical experiments. Briefly, the oxidation peak observed at +0.15 V is due to the generation of the soluble complex ${\mathrm{CuCl}}_{2}^{-}$ .^19,20^ This reaction is controlled by mass-diffusion and, due to the consumption of chloride, it is followed by the generation of insoluble CuCl, which remains attached to the electrode surface.^21,22^ In the anodic region, *E* > +0.20 V, the anodic dissolution of the surface is observed, involving the oxidation of the Cu-PCE leading to the release of Cu^2+^.^23,24^ Next, in the backward scan, between +0.40 and 0.00 V, the reduction of Cu­(II) cations together with the soluble chloride complexes (e.g., ${\mathrm{CuCl}}_{2}^{-}$ ) and insoluble CuCl leads to the formation of SERS-active CuNPs. Adenine is expected to remain unmodified throughout the whole CV because its redox potential lies outside the investigated potential window.^25^

**Figure 2:**
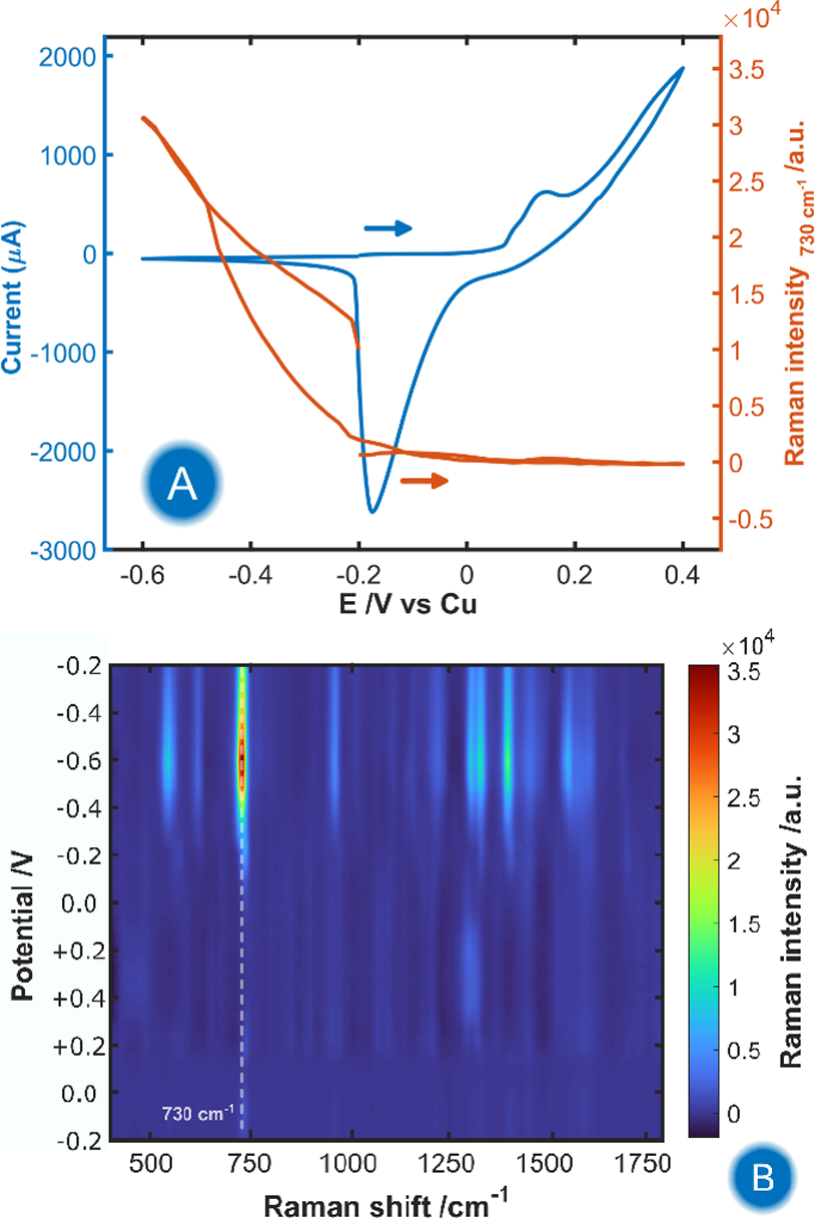
(A) CV and CVR at 730 cm^–1^ of 8 μM adenine in 0.1 M HCl during the roughening of the Cu-PCE. (B) Contour plot corresponding to the evolution of Raman spectra acquired during the experiment. The integration time of the Raman spectra was 1 s. The potential was scanned from −0.20 to +0.40 V and to −0.60 V. Scan rate was 0.02 V/s.

The Raman spectroscopic results are summarized in Figure 2B, which shows the evolution of the Raman spectra recorded along the CV. The most significant spectral changes were observed in the cathodic region (*E* < −0.20 V, backward scan), where the evolution of several Raman bands was observed, reaching their maximum intensity around −0.60 V. The Raman spectra observed in the cathodic region correspond to adenine (Figure S2) adsorbed on the CuNPs electrogenerated after reduction of CuCl, ${\mathrm{CuCl}}_{2}^{-}$ and Cu^2+^. The assignments of the recorded Raman bands to those reported in the literature are shown in Table S1.

The evolution of the adenine Raman intensity is best followed at 730 cm^–1^ as a function of potential, as shown in Figure 2A (orange line). This representation is known as Cyclic VoltaRamangram (CVR).^26^ This curve shows that the Raman signal of adenine is not detectable in the anodic region (*E* > −0.20 V). After the reduction of CuCl (−0.20 V), the Raman intensity increases due to the electrosynthesis of SERS-active CuNPs. The Raman enhancement is more noticeable at more negative potentials due to the stronger adsorption of adenine on CuNPs.^27^ These results demonstrate that the developed Cu-PCE allows the SERS detection of 8 μM adenine in a relatively fast experiment (100 s), obtaining a signal-to-noise ratio (Figures 2B and S2) that allows us to detect this molecule at micromolar level easily, thus competing with the sensitivity of other high-ordered Cu SERS substrates.^28^ The estimated analytical enhancement factor (AEF) for this CuNPs substrate is 3.6 × 10^6^. Although it is possible to carry out the ex-situ SERS detection of adenine, we found this operando methodology to be more sensitive and useful for quantitative analysis, due to the wealth of information provided by the dynamic evolution of the Raman intensity during the experiment.

To test the limits of the proposed methodology, we performed a Raman-SEC study of adenine adsorption on Cu-CPE over 5 ORCs in 0.1 HCl. We found that the use of Cu-PCE electrodes allows to perform an ultrafast characterization of adenine behavior, even at low concentrations (100 nM) and high scan rates (0.2 V/s). The results are summarized in Figure S3. The integration time of the Raman spectra was set to 20 ms to obtain a suitable temporal resolution at this high scan rate. High temporal resolution helped us to identify fast changes in adenine adsorption during the experiment. A blue shifting of 11 cm^–1^ in the vibrational band assigned to the ring breathing mode of adenine (730 cm^–1^) can be clearly observed when potential is scanned in anodic region (Figure S3C), which could be related to a change in adsorption geometry or maybe to changes in the tautomeric form of adenine.^29^

In summary, Cu-PCEs can easily be used to generate Cu-SERS substrates. Moreover, the CuNP substrate quality is sufficient to achieve nanomolar detection of adenine, even using integration times of 20 ms, which is significantly lower than those typically found in the literature.

### 3.3 Cu-PCE for the Detection of Tertiary Amines

To further evaluate the use of Cu-PCE as a SERS platform, the Raman response of three different bioactive amines (fentanyl, cocaine and paraquat) was analyzed during roughening of the Cu substrate. Fentanyl and cocaine are two widely used drugs of abuse, while paraquat is an herbicide strictly forbidden in the EU due to its acute toxicity, but still commonly used in countries like India.^30,31^

An experiment similar to that shown in Figure 2 was performed using the new analytes. The results are summarized in Figures 3 and S4. The orange lines in Figure 3 represent the CVR of the most intense band for each analyte. Raman enhancement of the three analytes can be clearly seen during the experiment, even at very low concentrations (e.g., 1 μM fentanyl or 30 nM paraquat). Interestingly, the three molecules exhibited different behaviors from those exhibited by adenine. In this case, a small Raman enhancement was observed in the anodic scan after the formation of CuCl on the electrode surface (*E* > +0.20 V). Nevertheless, the maximum Raman enhancement was observed in the backward scan for all analytes during the reduction of insoluble CuCl on the WE surface, around −0.20 V. In this maximum enhancement, the AEF was estimated to be around 3 × 10^8^, 2 orders of magnitude greater than the AEF of CuNPs for adenine. No significant Raman enhancement was observed after this reduction peak, centered around −0.10 V, as was observed for adenine (Figure 2). Only fentanyl exhibits a modest Raman signal enhancement at potentials below −0.20 V (Figure 3A, orange line), suggesting potential-favored adsorption of this analyte on metallic CuNPs. It is noteworthy that the good performance of the Cu-PCEs proposed in this work, to detect compounds at very low concentrations. These results provide new insights into EC-SERS analysis with Cu electrodes, rather unusual substrates in the literature.

**Figure 3:**
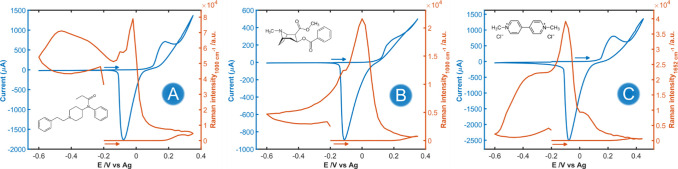
CV (blue lines) and CVR (orange lines) of (A) 1 μM fentanyl at 1000 cm^–1^, (B) 50 μM cocaine at 1000 cm^–1^, and (C) 30 nM paraquat at 1652 cm^–1^ during an ORC of Cu-PCE in 0.1 M HCl. Potential was scanned from (A, B) −0.20 V to +0.35 and −0.60 V and (C) −0.20 to +0.45 to −0.60 V, starting the experiment through the anodic direction. Integration time was 1 s. Scan rate was 0.02 V/s.

The behavior of the Raman signal of these tertiary amines during the CV experiments suggests that the main structure involved in the Raman enhancement are not plasmonic CuNPs, since the Raman enhancement occurs before the reduction of CuCl.

This behavior is similar to the EC-SOERS phenomenon,^26,32–34^ a Raman enhancement strategy reported in other works that involves the generation of semiconductor or dielectric nanostructures on the electrode surface, such as AgCl or CuI, during the electrochemical oxidation of a metal electrode (typically Ag or Cu electrodes). These structures can exhibit strong SERS activity but limited interactions with most analytes. During oxidation of the WE surface, metal cations (Ag^+^ and Cu^2+^) can be released. These cations can modulate the surface charge of the SERS-active semiconductor nanostructures, and facilitate the adsorption of negatively charged molecules.^35^ In this work, it is possible that we are observing the SERS properties of CuCl. Nevertheless, the analytes studied in Figure 3 are either neutral or positively charged, especially at low pH, where protonation of tertiary amines can occur. This difference with the previously reported EC-SOERS substrates suggests that the excess Cl^–^ present in these experiments promotes the adsorption of positively charged molecules.

Cu-PCE can be reused as an EC-SERS or EC-SOERS platform; however, its sensitivity diminishes with repeated use. Furthermore, the substrate exhibits such a high sensitivity that, usually, target molecules are detected even after cleaning processes, rendering these electrodes more suitable as disposable devices. It is imperative to develop additional strategies to effectively clean the SERS substrate without compromising the SERS enhancement quality. Nonetheless, this issue remains unresolved within the SERS community for any SERS substrate.

To the best of our knowledge, this is the first time that CuCl substrates have been described as SERS active. To shed more light on these experiments, we used SEM imaging and UV/vis-BSEC to investigate the processes taking place on the WE surface.

### 3.4 SEM Study of the Cu-PCE Electrode Surface

SEM characterization of the Cu-PCE surface was performed to obtain more information about the processes taking place during an ORC in 0.1 M HCl. Figure 4 shows the SEM images obtained at different potentials during the CV Raman-SEC experiment (Figure 4D).

**Figure 4:**
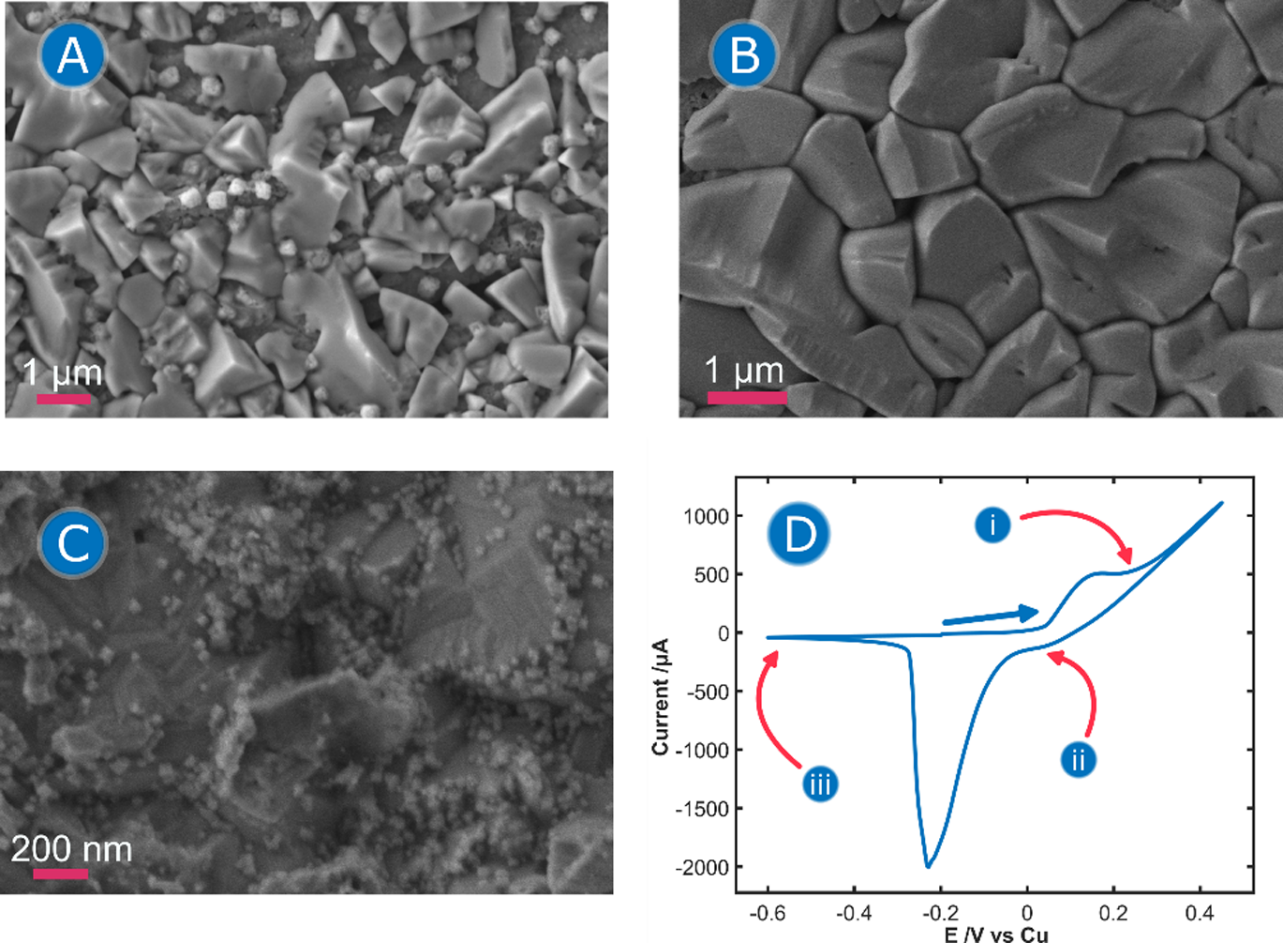
(A–C) SEM images at different potentials of a Cu-PCE during (D) ORC in 0.1 M HCl. Potentials were stopped at (A) +0.25 V, (B) +0.10 V and (C) −0.60 V. (D) Typical CV, pointing the potentials corresponding to each SEM image (i:A: +0.25 V, ii:B: +0.10 V, iii:C: −0.60 V). The potential was scanned from −0.20 to +0.45 V and to −0.60 V. Scan rate was 0.02 V/s. All samples were washed with 0.1 M HCl after the electrochemical experiment previous to SEM imaging, to avoid any chemical transformation.

SEM images confirm the formation of insoluble CuCl NPs on the Cu-CPE surface at +0.25 V, after the anodic peak at +0.20 V (Figure 4A). The observed crystals exhibit a variety of shapes, from pyramids to cubes. The size of these structures is inhomogeneous, varying from dozens of nanometers to several micrometers. During the further oxidation of the electrode, the growth of these crystals was observed (Figure 4B), reaching micrometric size and completely covering the surface of the electrode. EDX mapping confirmed that the structures formed during the anodization of Cu are CuCl structures (Figures S5 and S6, Tables S2 and S3). Finally, the application of cathodic potentials led to the reduction of CuCl, resulting in the formation of metallic CuNPs (Figure 4C) with an average size of 32 nm and a standard deviation of 10.7 nm. A histogram of these nanoparticles is provided in Figure S7.

Interestingly, we found that the CuCl structures shown in Figure 4A,B can be modified by washing with deionized water, resulting in the generation of cubic Cu oxide NPs (Figure S8), probably Cu_2_O.^36^ The SEM images obtained after washing with deionized water could lead to misleading conclusions, suggesting that these cubic nanostructures could be CuCl nanocrystals, as shown in Figure S5. To avoid this artifact, it is necessary to wash the electrode with the modification electrolyte (0.1 M HCl), which prevents the chemical transformation of the CuCl substrate. This observation illustrates the intrinsic limitations of ex-situ analysis methodologies compared to *operando* techniques, which can generate conclusions influenced by hidden external variables, like the reaction of the substrate with water in this case. *Operando* Raman spectra obtained during the washing of the sample confirm the transformation of the substrate (data not shown). To overcome these limitations, an *operando* analysis of the generation of the substrate was carried out by UV/vis-BSEC.

### 3.5 Bidimensional UV/Vis Absorption Spectroelectrochemistry Study of Cu-PCE

UV/vis-BSEC^37^ is a SEC technique in which two different SEC configurations (normal and parallel) are simultaneously used in the same experiment to interrogate the electrode–solution interface from two complementary points of view.

UV/vis absorption spectroelectrochemistry (UV/vis-SEC) can be performed in different configurations, such as normal or parallel. Although both configurations use UV/vis to interrogate the electrochemical system, each configuration provides different information. Normal configuration interrogates primarily the electrode surface, and parallel configuration only provides information about the solution adjacent to the electrode. In BSEC, both configurations are measured at the same time, providing a full characterization of the system.

Figure 5F shows a schematic representation of the BSEC experiment. To measure UV/vis-SEC in a parallel configuration, two bare optical fibers were attached to a 3D printed piece, as previously reported,^18^ and placed over the electrode. These optical fibers provide information about the chemical species present in the diffusion layer adjacent to the WE surface. Normal configuration UV/vis-SEC was carried out with a reflection probe focused on the WE. With the normal configuration, it is possible to obtain information about the processes taking place on the WE surface, and also in the solution adjacent to the electrode, since the perpendicular light beam also interrogates the solution. However, the sensitivity is much lower in normal configuration as the optical path-length is shorter than in parallel configuration.

**Figure 5:**
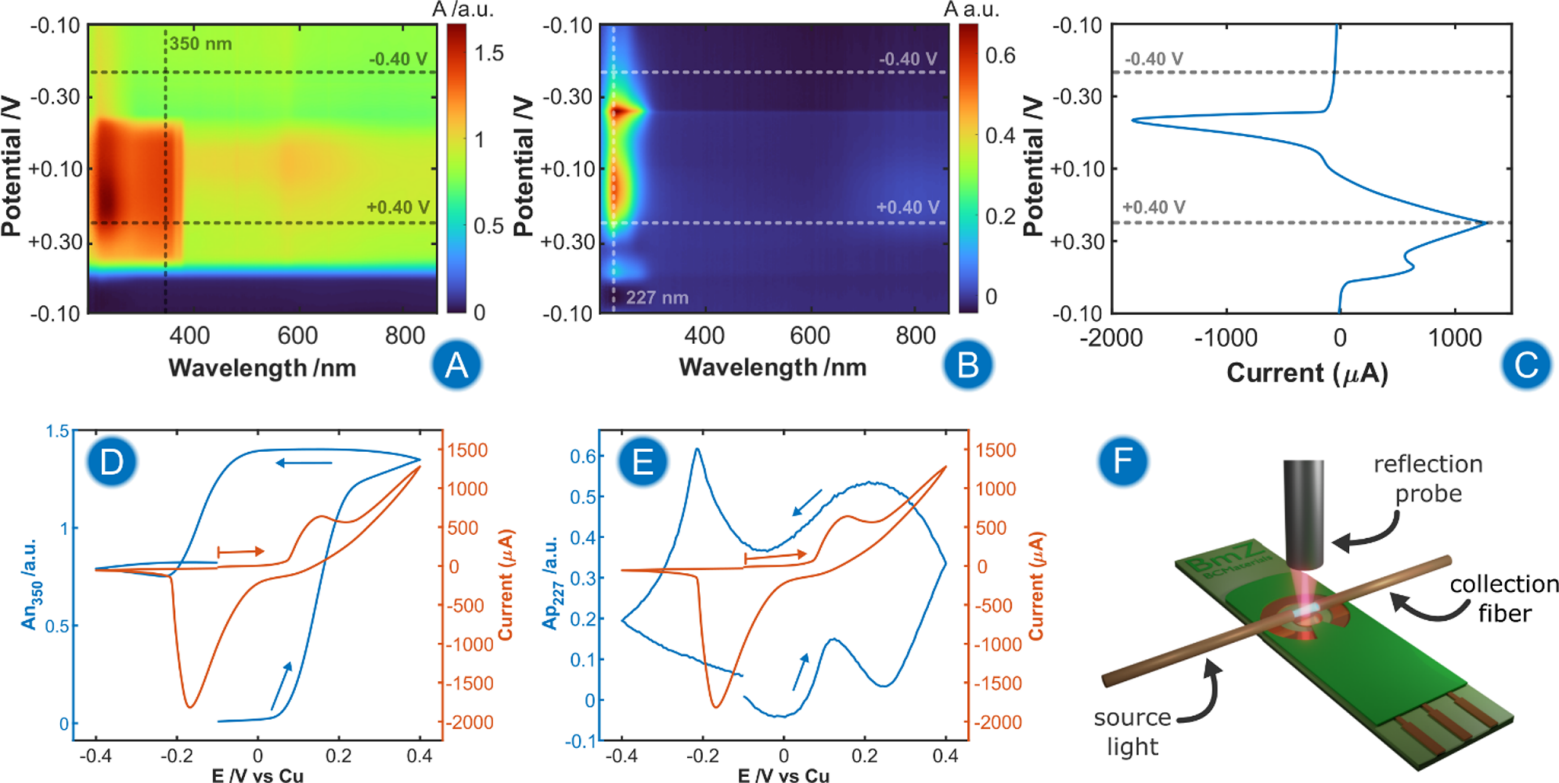
BSEC study of a Cu-PCE during roughening in 0.1 M HCl. Contour plots of UV/vis absorption spectra registered in the (A) normal configuration and (B) parallel configuration. (C) Potential vs current representation of the CV. (D) CVA at 350 nm in normal configuration (blue line) and CV (orange line). (E) CVA at 227 nm in parallel configuration (blue line) and CV (orange line). (F) Schematic of the experimental setup used for UV/vis-BSEC experiments. The potential was scanned from −0.10 to +0.40 to −0.40 V. Scan rate was 0.02 V/s. The reference absorption spectra were recorded at the beginning of the experiment. Integration times were 250 ms for the parallel configuration and 200 ms for the normal configuration.

The BSEC experiment enables the extraction of *operando* and synchronized information from both optical configurations in a single experiment. This multiresponse technique was applied to monitor changes occurring on Cu-PCE during the electrosynthesis of SERS substrates under the conditions discussed above. The use of Cu-PCE allows the rapid and straightforward preparation of a bidimensional setup with bare optical fibers and reflection probes, as shown in Figure 5F. A summary of the results is presented in Figure 5A–E.

Figure 5A,B represent the contour plots registered in normal and parallel configuration, respectively, where it represents the evolution of UV/vis absorption spectra in both configurations during a CV of a Cu-PCE in 0.1 M HCl. The potential vs current plot (Figure 5C) is provided to clarify the correlation between the recorded spectroscopic changes and the electrochemical signal.

In normal configuration (Figure 5A), changes in absorbance were observed at all registered wavelengths. Because the normal configuration provides information mainly about the processes occurring at the WE surface, these changes should be interpreted as changes in the electrode reflectivity. A well-defined broad absorption band, ranging from 250 to 370 nm, dominates the registered UV/vis absorption spectra between +0.10 and −0.10 V, corresponding to the absorption spectra of CuCl nanocrystals present on the Cu-PCE surface.

The evolution of the absorption bands can be better studied when a single wavelength is represented as a function of the applied potential. This representation is known as cyclic voltabsorptogram (CVA).^38^ Figure 5D shows the CVA at 350 nm for the normal configuration (blue line), which reveals that the absorption band of insoluble CuCl greatly increases at potentials higher than +0.10 V, which is correlated with the generation of CuCl structures on the surface (Figure 4A).

During the backward scan, the absorbance of CuCl reaches a maximum and stable value from +0.40 to 0.0 V (Figure 5D, blue line). Below 0.0 V, the absorbance at 350 nm decreases due to the reduction of CuCl, reaching final values of 0.8 a.u. from −0.20 V onward. The lack of recovery of the initial values of absorbance at 350 nm is due to the changes in the surface reflectivity caused by surface roughening, as shown in the SEM images, Figure 4A. However, correcting the absorbance at 350 nm relative to the absorbance at 800 nm, where no defined band is registered, reveals that the reduction of CuCl is fully reversible (Figure S9A,B). The derivative of the CVA at 350 nm in normal configuration revealed a good correlation between the electrochemical processes observed in the CV and spectroscopic information (Figure S9C), confirming that the absorption band between 250 and 370 nm corresponds to insoluble CuCl.

After analyzing the information provided by normal configuration, we now focus on the analysis of parallel configuration, which provides information about the chemical species in the diffusion layer (Figure 5B). In this configuration, a well-defined absorption band centered around 227 nm with a shoulder around 270 nm was observed, exhibiting maximum values after reaching the anodic vertex potential, +0.40 V. This band must be related to the presence of soluble Cu­(I) chloride complexes, such as ${\mathrm{CuCl}}_{2}^{-}$ , in the diffusion layer, complexes which have been reported to have absorption bands in this spectral region.^39^ It should be noted that the parallel configuration cannot detect surface changes because the thickness of the Cu structures is much smaller than the optical fiber diameter, 100 μm. Moreover, approximately the first 20 μm of the solution adjacent to the electrode cannot be interrogated in this configuration because of the cladding of the optical fibers.^40^

Figure 5E (blue line) shows the CVA at 227 nm in the parallel configuration. The absorbance at this wavelength shows a complex behavior that can be related to the relative concentrations of Cl^–^ and Cu^+^ in the proximity of the electrode. First, it was observed that this band reached a local maximum at +0.12 V, indicating the generation of ${\mathrm{CuCl}}_{2}^{-}$ complex in solution. At more anodic potentials, the absorbance at 227 nm decreases until +0.25 V. This is consistent with the literature, which suggests that the first step in the oxidation of Cu electrodes in chloride media involves the generation of ${\mathrm{CuCl}}_{2}^{-}$ soluble complex, followed by the generation of insoluble CuCl, when the local chloride concentration decreases.^19,20,41^ These observations provided by BSEC experiments confirm by a direct detection of the spectrum of this soluble complex, the results based on electrochemical measurements.^19,20,41^

A continuous increase of the absorbance in parallel configuration at 227 nm is observed from +0.30 V to the anodic vertex potential, and from +0.40 to +0.20 V in the backward scan (Figure 5E, blue line), due to the release of Cu­(I) and Cu­(II) to the solution, which generates higher concentration of soluble complexes on the diffusion layer of the WE. However, this increase in absorbance was only observed during the oxidation of the Cu electrode. Otherwise, a slight decrease in absorbance was observed at potentials below +0.20 V in the backward scan due to the diffusion of soluble complexes into the bulk solution and to the reduction of Cu­(II). The band at 800 nm observed in parallel configuration, which is associated with Cu­(II), allows us to distinguish between Cu­(II) and Cu­(I). Finally, a sharp increment of absorbance at 227 nm at −0.20 V is registered during the reduction of CuCl present on the Cu-CPE surface. This peak in absorbance suggests that a high concentration of Cl^–^ and Cu^+^ ions are liberated during the reduction of CuCl, which latter forms high concentration of soluble Cu chloride complexes (e.g., ${\mathrm{CuCl}}_{2}^{-}$ , ${\mathrm{CuCl}}_{3}^{2-},{\mathrm{CuCl}}_{4}^{3-}$ ), releasing an excess of Cl^–^ to the solution, which also helps to generate the complexes. This interesting observation is in good agreement with the results of Cherevko group, obtained using ICP-MS coupled with electrochemistry, during the study of the reduction of metal oxides.^42,43^ This group has reported for many years that during the electrochemical reduction of metallic oxides and salts, great amounts of metallic cations are released to the adjacent solution, revealing the complexity of electrochemical ORC procedures. UV/vis-BSEC outperforms ICP-MS in terms of speciation of the compounds formed during the releasing to the solution.

Notably, the increase in absorbance at 227 nm during CuCl reduction arises approximately concomitantly with the maximum Raman enhancement observed for many tertiary amines (Figure 3). From −0.20 V onward, and until the end of the experiment, the absorbance in the parallel configuration decreased because of the reduction of all Cu species to CuNPs.

Our group has previously reported a strong correlation between the surface charge of several semiconductor/dielectric materials and their interaction with different molecules, which would result in a significant enhancement of the Raman signal due to the presence of adsorbates.^35^ BSEC results provide information about the local concentration of both Cu cations and Cl^–^ in the diffusion layer, an important step to study the role of these ions in the Raman enhancement observed during CuCl reduction.

## 4 Conclusions

Cu-PCB materials were used to fabricate a three-electrode electrochemical platform made entirely of Cu. This Cu-PCE platform has been used for the electrosynthesis of several Raman-enhancing substrates for a variety of molecules, including biomarkers, drugs, and herbicides. Nevertheless, it can be expected that this substrate could be used to detect many other types of molecules. The sensitivity of Cu-PCE electrodes has been demonstrated to be highly effective, enabling the detection of molecules at very low concentrations. However, to expand their utility for quantitative applications, improvements in reproducibility are needed. Further investigations are required to examine the type of PCB employed and the protection of the Cu surface prior to its utilization.

The use of PCB-Cu materials has facilitated the performance of Raman and bidimensional SEC experiments which would otherwise be laborious and time-consuming.

Raman-SEC experiments revealed that PCB-Cu substrates are an interesting approach to carry out the Raman detection of tertiary amines, especially when using CuCl-based SERS substrates. BSEC experiments revealed that the high sensitivity of these CuCl-based substrates could be related to the accumulation of a high concentration of ions in the proximity of the electrode, which could lead to electrostatic interaction between the analytes and the SERS substrates. Nevertheless, this increase in the Raman signal during the reduction of CuCl deserves further study because a combination of the EC-SERS and EC-SOERS phenomena could occur during this electrochemical process.

## Supplementary Material



## Notes

Detailed description of the manufacturing of Cu-PCE, voltammetric study of Cu-PCE stability, Raman assignment of adenine, further explanation of adenine SERS spectra at high scan rates (0.2 V/s), EDX mapping of the CuCl structures formed during anodization of Cu-PCE, histogram of the size of CuNPs formed after reduction of CuCl, EDX and SEM study of the copper oxide nanocubes formed after washing the CuCl substrates with deionized water, contour plots of fentanyl, cocaine, and paraquat during ORC of a Cu-PCE, and further details of the BSEC study of the Cu surface during ORC (PDF)
