# Supplementary material for: Disposable Copper Electrodes Based on Printed Circuit Board Technology for the Operando Generation of Raman-Enhancing Substrates
Source: Anal Chem. 2025 Sep 16;97(38):20753–60. doi: 10.1021/acs.analchem.5c02381 (PMC12489897; doi:10.1021/acs.analchem.5c02381)
Supplement: Supplementary file 1 [file ac5c02381_si_001.pdf]

Supporting information:

Disposable Copper Electrodes Based on Printed Circuit Board Technology for Operando Generation of Raman-Enhancing Substrates.

Martin Perez-Estebanez[a], Luis Romay[a], Maria Huidobro[a], Pello Nuñez-Marinero[b], Aranzazu Heras[a], F. Javier del Campo[b]*, Alvaro Colina[a]*

[a] Department of Chemistry, Universidad de Burgos, Pza. Misael Bañuelos s/n, E-09001, Burgos, Spain.

[b] BCMaterials, Basque Center for Materials, Applications and Nanostructures. UPV/EHU Parque Científico, E-48940, Leioa, Bizkaia, Spain.

* Corresponding authors: javier.delcampo@bcmaterials.net, acolina@ubu.es

Index

Manufacture process of Cu-PCE 2
Cu-PCE stability test in basic medium 3
Raman assignment of Adenine spectrum 4
Raman-SEC study of adenine with 20 ms acquisition time 5
Raman spectroelectrochemistry analysis of tertiary amines 7
EDX mapping of CuCl structures 8
Histogram of CuNPs 10
Evolution of CuCl structures to Cu oxide nanocubes 11
Bidimensional UV/Vis absorption spectroelectrochemistry study of Cu-PCE 13
References 14

Manufacture process of Cu-PCE

Cu-printed circuit electrodes (Cu-PCE) were fabricated through a combination of screen printing and wet etching of printed circuit boards (PCB). First, all PCB boards were cut to 100 mm x 100 mm plates and registration marks were milled using a desktop CNC milling machine (Roland DG MDX-50).

Following cutting and marking, the boards were thoroughly cleaned in a sequence of baths containing acetone, isopropanol, and Milli-Q water. Using an ultrasound bath could enhance the effectiveness of this cleaning process and ensure the removal of all residues from the Cu surface. Note that some boards came pre-coated with a photoresist layer to facilitate patterning using film positives. To ensure that all boards underwent the same process, removal of this photoresist layer in the acetone cleaning bath was assisted by a previous 3-minute flood-exposed under a 500W UV (Hg) lamp and development using Microtrack developer as per manufacturer instructions (Microtrack, RS-Amidata). Next, the clean boards were dried under air stream.

The final PCE design was screen-printed using resist layer (XZ77 Blue plating resist, Sun Chemicals, Servilan) defining the desired Cu parts on the clean surface of the Cu-PCB plates. After curing this resist, the PCBs were immersed in a $FeCl_3$ bath (Multicomp, Farnell) where the exposed Cu areas were chemically etched. To ensure homogeneous etching across all areas, the bath containing the boards was gently rocked on an SK_R1807-E shaker. Etching times may vary based on solution condition, plate size, and Cu content. A10 cm x 10 cm plate with a 35 μm Cu layer typically takes 20-30 min in a fresh solution. Following chemical etching, the resist was stripped off in a 5% NaOH solution, followed by the same cleaning process described above, with subsequent baths in acetone, isopropanol, and Milli-Q water. Finally, a dielectric layer was printed to protect the tracks and define the electrode areas (LOCTITE® EDAG 452SS, Tetrachim).

Each PCB board contained up to 14 individual electrodes that were cut out by milling using micro-toothed end bits in the milling machine. Subsequently, each electrode was cleaned and vacuum-sealed with RP-1AN humidity and oxygen traps (Mitsubishi Chemical Company).

Cu-PCE stability test in basic medium

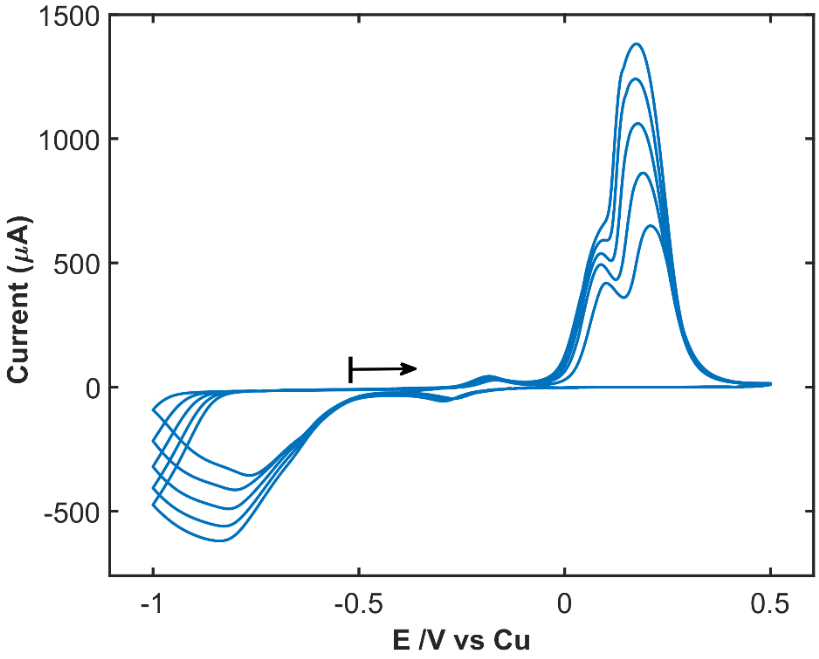

Figure S1. Five consecutives CVs of a Cu-PCE electrode in 1 M NaOH

Raman assignment of Adenine spectrum

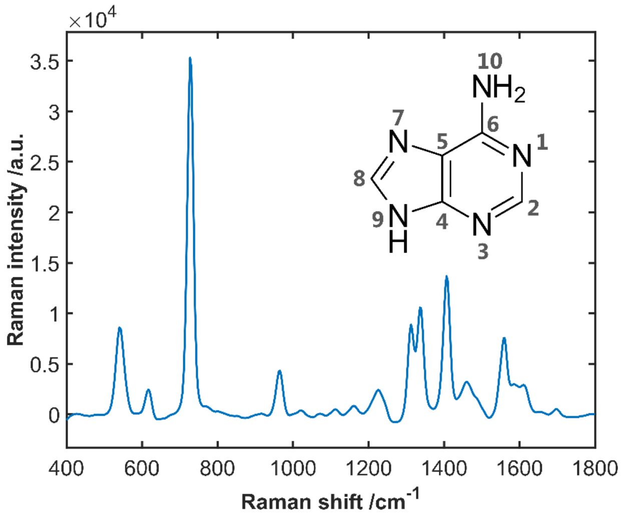

Figure S2. Raman spectrum of 8 µM adenine in 0.1 M HCl registered at -0.60 V during a Raman SEC experiment on a Cu PCE. Inset: chemical structure and atomic labelling of adenine. The electrochemical conditions were identical to those shown in Figure 2.

Figure S2 shows the Raman spectra of adenine 8 µM in 0.1 M HCl during the ORC of a Cu-PCE. This spectrum was obtained at -0.60 V from the experiment shown in Figure 2, in the main text. It is performed a comparison of the Raman bands registered in this experiment with the adenine spectra reported in literature to confirm that the observed spectrum corresponds to adenine (Table S1).

TABLE S1. BAND ASSIGNMENT OF ADENINE SERS SPECTRA[1–3].

EXPERIMENTAL SERS SPECTRA (cm^{-1})	CALCULATED SERS SPECTRA (cm^{-1}) [1–3]	Band Assignment
**545**	534-536	in/out-plane def. R6 (sqz group N1-C6-C5, C2-N3-C4)
**619**	619-623	in-plane def. R6 (sqz group C4-C5-C6, N1-C6-N10) and R5 (sqz group C5-N7-C8)
**730**	722-723	ring breathing
**967**	939-942	in-plane def. R5 (sqz group N7-C8-N9); rock. NH2; str. N1-C6
**1315**	1306-1308	in-plane str. C2-N3, N1-C2, C5-N7, C5-C6
**1341**	1331-1333	in-plane str. C6-N1, C8-N9, N3-C4, C5-N7, N1-C2
**1410**	1417-1419	in-plane str. C4-N9, C4-C5, C6-N10, C7-C8; in-plane bend. C2-H
**1562**	1596-1597	in-plane str. N3−C4, N1−C6, C2−N3; in-plane sciss. NH_2

R6: six-membered ring, R5: five-membered ring; bend.: bending; def.: deformation; rock.: rocking; sciss.: scissoring; sqz.: squeezing; str.: stretching;

Raman-SEC study of adenine with 20 ms acquisition time

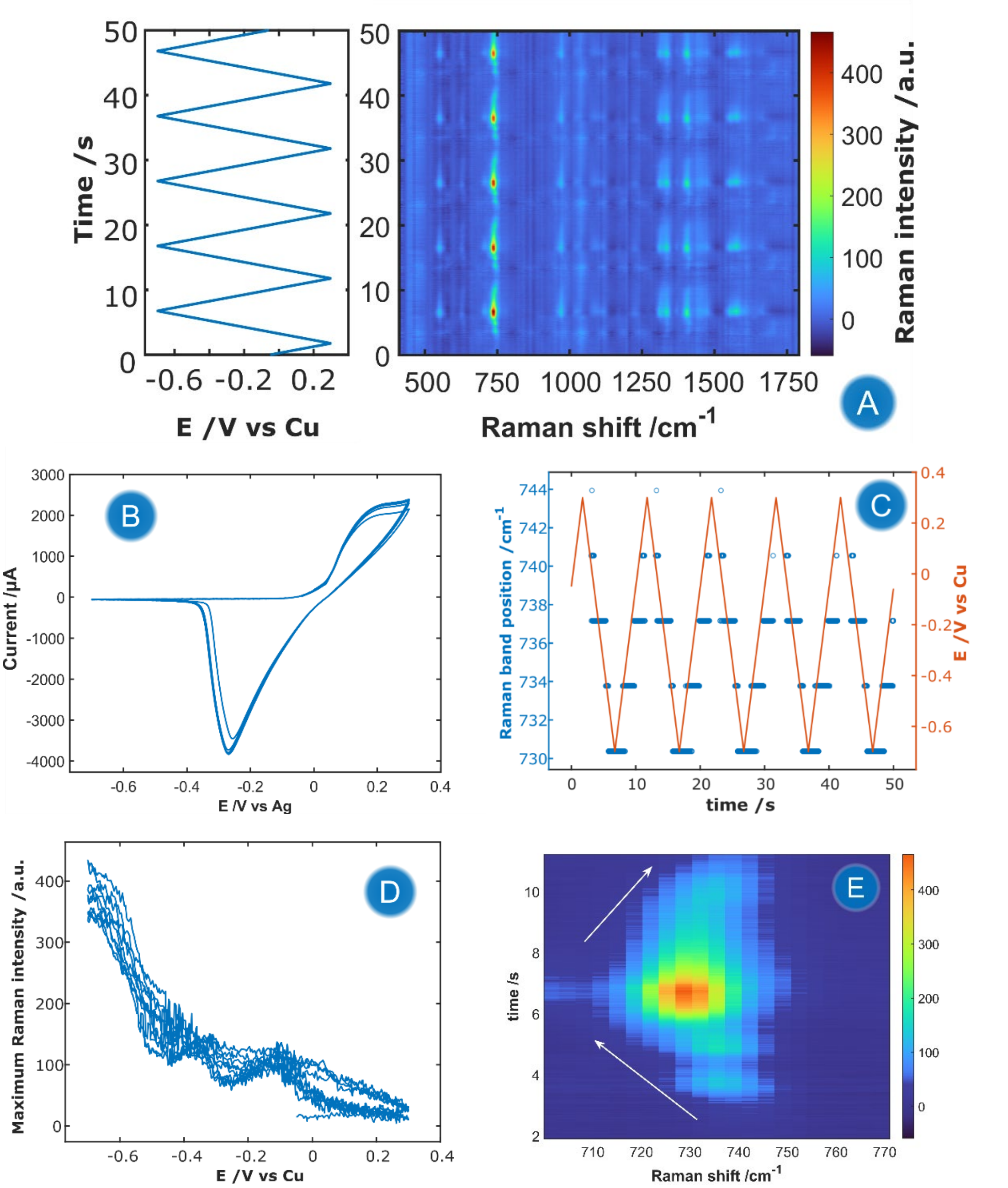

Figure S3. Raman-SEC study with a Cu-PCE of 100 nM adenine in 0.1 M HCl during 5 ORCs. (A) Contour plot of the Raman spectra, (B) CV, (C) evolution of the position of the Raman band at 730 cm^{-1} (only peaks with intensities greater than 50 a.u. were considered), and (D) CVR of the Raman band around 730 cm^{-1}, corrected with the observed shifting during the experiment. (E) Detailed contour plot of the Raman spectra during the first cycle to observe the shifting of the Raman band. The integration time was 20 ms. 5 potential scans from -0.05 V to +0.30 V and to -0.70 V were applied. Scan rate was 0.2 V/s.

Figure S3A summarizes the Raman-SEC study of adenine during the performance of several ORCs on a Cu-PCE at high scan rates (0.2 V/s) and low acquisition time, showing all the Raman spectra recorded during the experiment. The change of scan rate from 0.02 V/s (Figure 2) to 0.2 V/s did not greatly influence the CV shape (Figure S3B), but lowered its intensity 10 times. The main processes observed in the CV are the oxidation of the Cu-PCE to form CuCl and $CuCl_2^-$, and the reduction in the backward scan to form CuNPs, as discussed in the main text. In this experiment, the maximum applied potential was +0.30 V, which is

lower than the experiment shown in Figure 2, to avoid excessive oxidation of the surface during the different potential cycles.

The high time resolution obtained during the experiment allowed for fine characterization of the behavior of the adenine Raman bands. For example, we were able to evaluate the shifting of the peak during the experiment with high precision (Figure S3C), which demonstrates that the ring-breathing mode of adenine, centered around 730 cm^{-1}, shifts significantly with the applied potential. It was observed that at anodic potentials, this mode experienced noticeable blue-shifting (higher Raman shifts), acquiring values up to 741 cm^{-1} in the most extreme cases. Furthermore, this blue shifting was reversible during the five registered ORCs, recovering the initial position at 730 cm^{-1} in the cathodic vertex potential (-0.70 V). A detailed image of the Raman band shifting can be observed in Figure S3E, corresponding to a detailed contour plot of the Raman band at 730 cm^{-1} during the first potential cycle

The observed shifting could be associated with changes in the adsorption behavior of adenine, which can vary with the applied potential. Another evidence for this is the change in the Raman intensity of adenine during the experiment. Figure S3D shows the Cyclic VoltaRamangram (CVR) of the ring breathing mode of adenine, at 730-740 cm^{-1}. It is observed that the largest Raman enhancement occurs at potentials <-0.40 V, where the reduction of CuCl species on the electrode surface was completed. However, Raman enhancement decreased rapidly when the potential was scanned in the positive direction. This experiment demonstrates how adsorption processes are strongly affected by the potential and the dynamics of these processes.

Raman spectroelectrochemistry analysis of tertiary amines

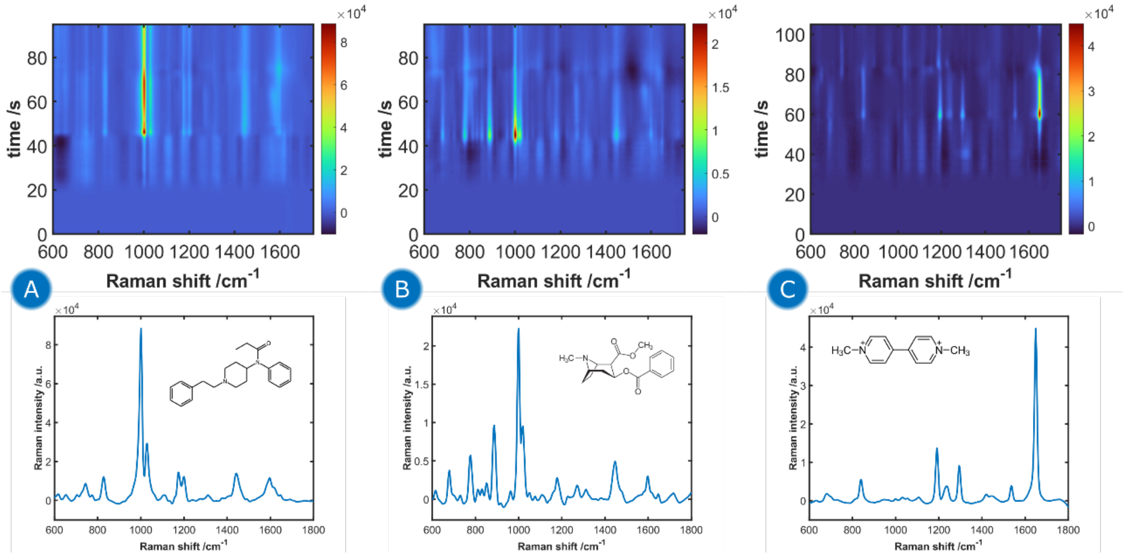

Figure S4. Contour plots and Raman spectra at the potential where the maximum Raman response is registered, acquired during an ORC of a Cu-PCE in 0.1 M HCl in the presence of (A) 1 µM fentanyl, (B) 50 µM cocaine, and (C) 30 nM paraquat. The experimental conditions were the same as those in Figure 3.

EDX mapping of CuCl structures

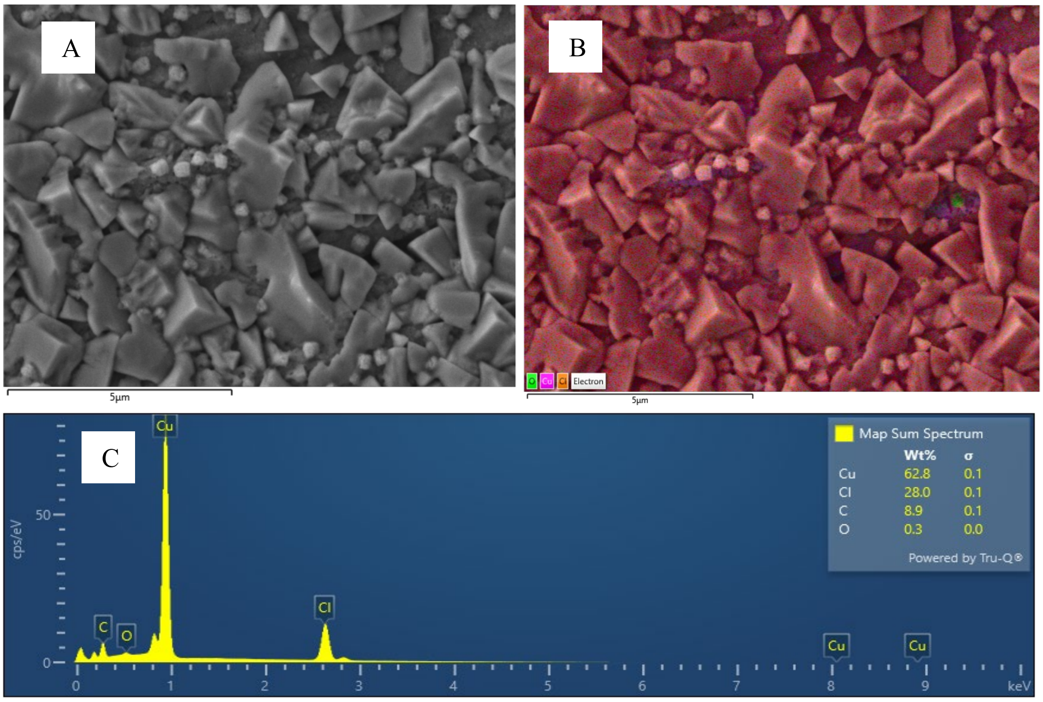

Figure S5. (A) SEM image, (B) EDX mapping and (C) total EDX spectrum of the sample formed after oxidation of a Cu-PCE in 0.1 M HCl stopping the potential at +0.25 V (point i in Figure 4D). Experimental conditions were the same as in Figure 4 in the main text: the potential was scanned from -0.20 V to +0.45 V and to -0.60 V. Scan rate was 0.02 V/s. Sample was washed with 0.1 M HCl previous to SEM and EDX analysis to avoid any chemical transformation.

Table S2. Atomic percentage of the CuCl nanostructures analyzed in Figure S5.

Element	Atomic %
C	29.15
O	0.71
Cl	31.15
Cu	38.99
**Total**	**100.00**

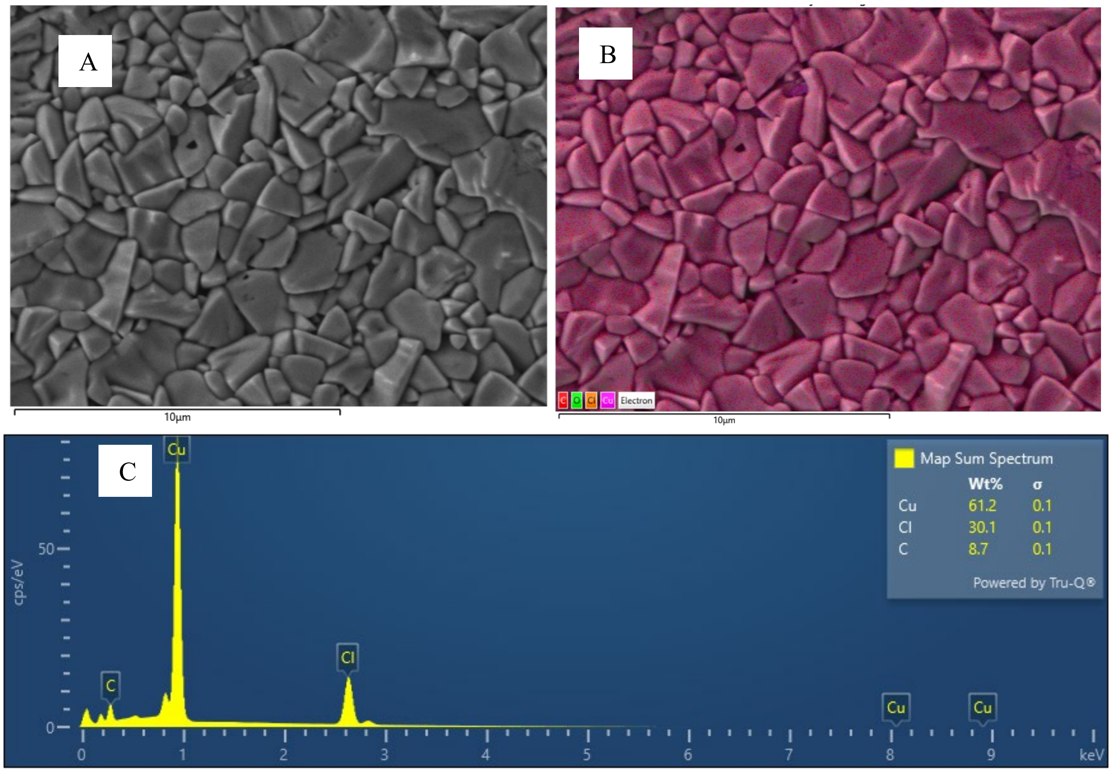

Figure S6. (A) SEM image, (B) EDX mapping and (C) total EDX spectrum of the sample formed after oxidation of a Cu-PCE in 0.1 M HCl stopping the potential at +0.10 V in the backward scan (point ii in Figure 4D). Experimental conditions were the same as in Figure 4 in the main text: the potential was scanned from -0.20 V to +0.45 V and to -0.60 V. Scan rate was 0.02 V/s. Sample was washed with 0.1 M HCl previous to SEM and EDX analysis to avoid any chemical transformation.

Table S3. Atomic percentage of the CuCl nanostructures analyzed in Figure S6.

Element	Atomic %
C	28.57
Cl	33.49
Cu	37.94
**Total:**	**100.00**

Figures S5 and S6 show the EDX mapping, EDX spectrum and atomic percentage extracted from the total EDX spectrum for the nanostructures formed during oxidation of a Cu-PCE in 0.1 M HCl. The EDX maps were performed in the same samples represented in Figures 4A and 4B in the main text.

The EDX analysis evidences that the chemical composition of the observed structures is CuCl, since the calculated atomic percentages of chloride and Cu are similar for both samples, Table S2 and S3. A slight Cu enrichment is observed for both samples, since these structures are located on a metallic Cu substrate. Thus, due to the penetration power of the electrons used for analysis, an enrichment is observed due to the contribution of the metallic substrate. For this reason, both the atomic and weight percentages obtained by EDX in these experiments should be considered qualitatively.

Histogram of CuNPs

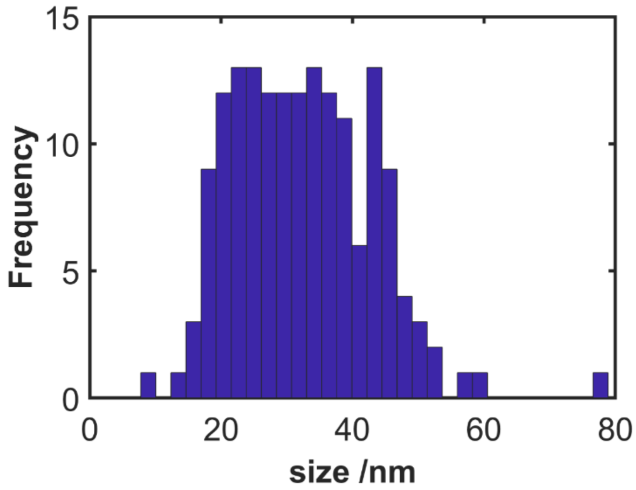

Figure S7. Histogram of the metallic CuNPs generated after applying an ORC of a Cu-PCE in 0.1 M HCl. Potential was scanned from -0.20 V to +0.45 V and to -0.60 V, finishing at -0.60 V. Scan rate was 0.02 V/s. Experimental conditions were the same as in Figure 4C.

Figure S7 shows the histogram of the CuNPs generated after reducing the CuCl formed on the Cu-PCE during its oxidation in 0.1 M HCl. The average size of the studied nanoparticles was 32.2 nm (n=164), with a standard deviation of 10.7 nm.

Evolution of CuCl structures to Cu oxide nanocubes

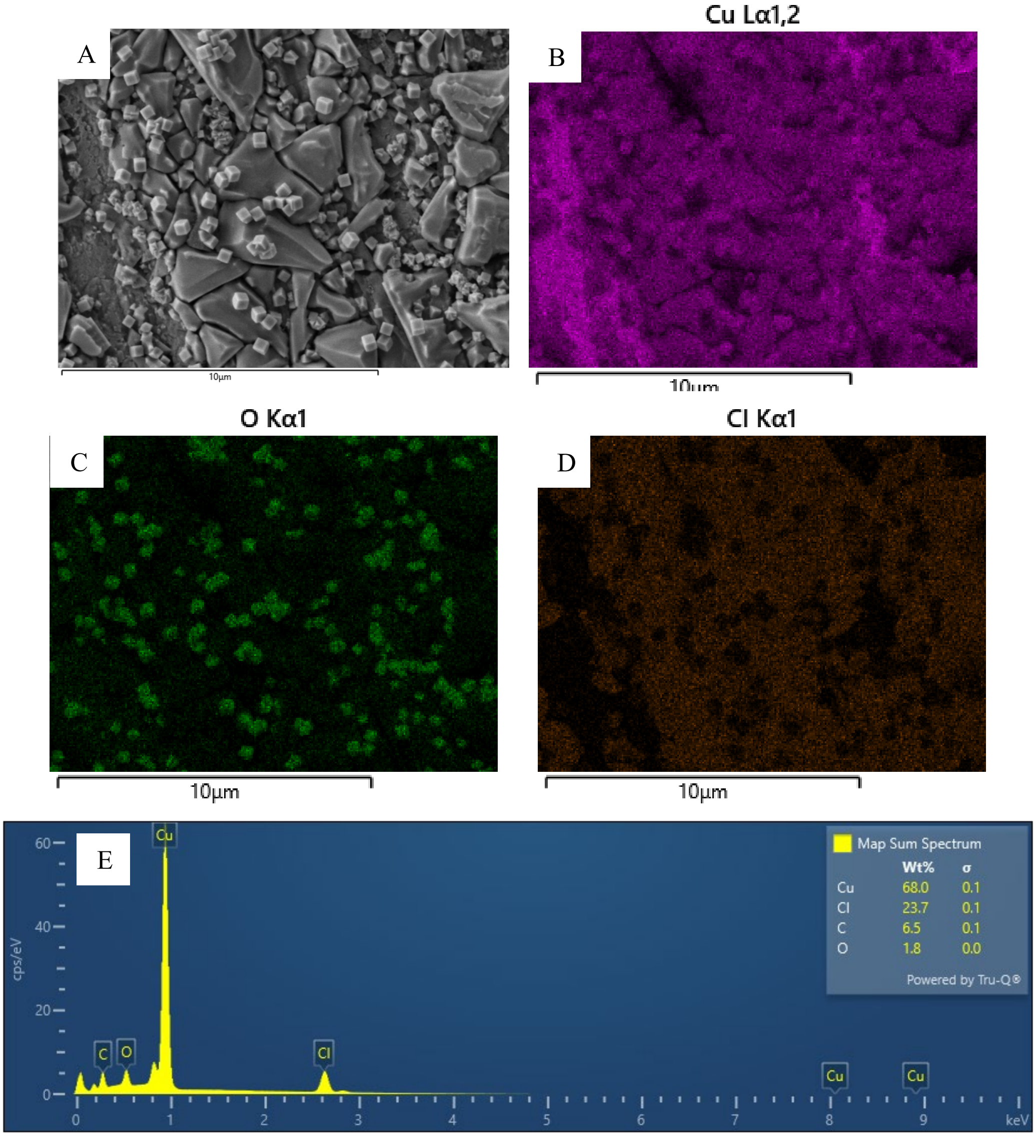

Figure S8. (A) SEM image, EDX mapping for (B) Cu, (C) oxygen and (D) chloride and (E) total EDX spectrum of the sample formed after oxidation of a Cu-PCE in 0.1 M HCl applying an LSV from -0.20 V to +0.25 V at 0.02 V/s. Sample was washed with deionized water previous to SEM and EDX analysis.

Figure S8 represents the SEM and EDX study of a Cu-PCE after oxidation in 0.1 M HCl. The electrochemical conditions of this sample were identical to the samples shown in Figure 4A and Figure S4. Nevertheless, there is a key difference among them: in Figure S7, the sample was washed with water previous to the SEM study, while the samples in Figures 4 and S4 were washed with 0.1 M HCl.

Figure S8 represents how the CuCl substrate generated during oxidation of Cu electrodes is dramatically transformed when it is in contact with deionized water, leading to the formation of well-defined nanocubes. EDX analysis clearly reveals that the chemical composition of the cubic particles is Cu oxide, probably Cu_2O which typically crystallizes in cubic form.[4] Operando Raman SEC revealed the apparition of oxide bands after oxidation of the electrode, when the electrolyte was changed from 0.1 M HCl to deionized water (data not shown).

These results highlight the relevance of using operando methodologies for the analysis of electrochemical substrates, which could be chemically unstable and can undergo chemical transformations that alter the conclusions of ex-situ analysis. At the same time, it shows a methodological error that could be present in many reported works, since the anodization of Cu in chloride media is a methodology typically used as precursor for the synthesis of Cu_2O nanocubes.

Bidimensional UV/Vis absorption spectroelectrochemistry study of Cu-PCE

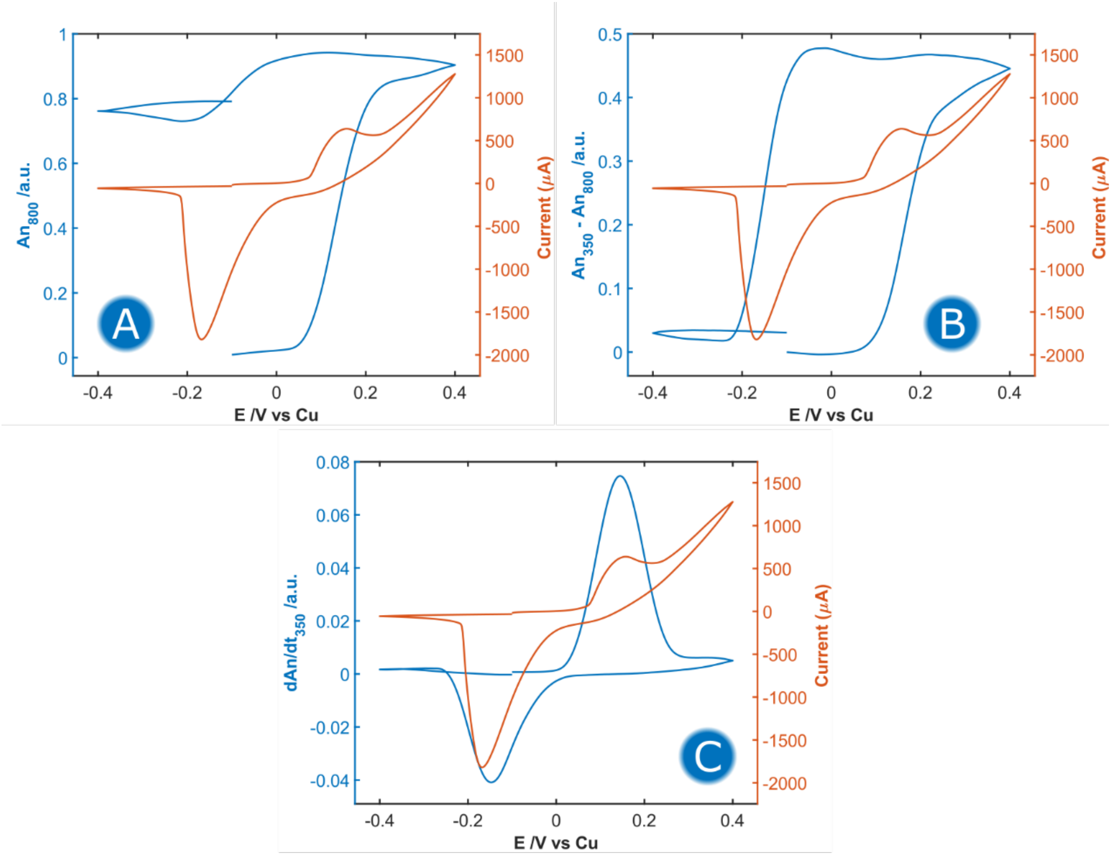

Figure S9. BSEC study of a Cu-PCE during roughening in 0.1 M HCl. (A) CVA at 800 nm in the normal configuration. (B) CVA at 350 nm in the normal configuration corrected by absorbance at 800 nm to determine the reflectivity changes that occurred on the WE surface by the roughening process. (C) DCVA at 350 nm in the normal configuration. CV is included in all figures for an easier comparison. The experimental conditions were the same as those shown in Figure 5.

References

(1) Yao, G.; Zhai, Z.; Zhong, J.; Huang, Q. DFT and SERS Study of 15N Full-Labeled Adenine Adsorption on Silver and Gold Surfaces. *J. Phys. Chem. C* **2017**, *121* (18), 9869–9878. https://doi.org/10.1021/acs.jpcc.7b00818.

(2) Papadopoulou, E.; Bell, S. E. J. Structure of Adenine on Metal Nanoparticles: pH Equilibria and Formation of Ag^+ Complexes Detected by Surface-Enhanced Raman Spectroscopy. *T J.Phys. Chem. C* **2010**, *114* (51), 22644–22651. https://doi.org/10.1021/jp1092256.

(3) Giese, B.; McNaughton, D. Surface-Enhanced Raman Spectroscopic and Density Functional Theory Study of Adenine Adsorption to Silver Surfaces. *J. Phys. Chem. B* **2002**, *106* (1), 101–112. https://doi.org/10.1021/jp010789f.

(4) Perales-Rondon, J. V.; Rojas, D.; Gao, W.; Pumera, M. Copper 3D-Printed Electrodes for Ammonia Electrosynthesis via Nitrate Reduction. *ACS Sustain. Chem. Eng.* **2023**, *11* (18), 6923–6931. https://doi.org/10.1021/acssuschemeng.2c06851.
